# Do Shifts in Honeybee Crop Microbiota Enable Ethanol Accumulation? A Comparative Analysis of Caged and Foraging Bees

**DOI:** 10.1007/s00248-025-02627-9

**Published:** 2025-12-04

**Authors:** Weronika Antoł, Bartłomiej Surmacz, Monika Ostap-Chec, Daniel Stec, Krzysztof Miler

**Affiliations:** Institute of Systematics and Evolution of Animals of the Polish Academy of Sciences, Kraków, Poland

**Keywords:** Foregut, Caged bees, Microbiome, Fermentation, Auto-brewery, Alcohol

## Abstract

Honeybees encounter low environmental doses of ethanol, primarily through fermenting nectar, which can have both beneficial and detrimental effects on their functioning. Yet, ethanol traces can also be detected in the crop of caged bees with no access to environmental food sources. This raises the possibility that endogenous ethanol accumulation could occur under restricted conditions, with microbial contributions as a potential mechanism. The crop microbiota, although less diverse than that in other gut segments, plays important roles in food fermentation and pathogen defense. We hypothesized that captivity-induced shifts in crop microbiota may facilitate fermentation, resulting in measurable ethanol. To test this, we compared the crop contents of naturally foraging hive bees and caged bees reared without access to the natural environment. Ethanol levels were low in both groups and did not differ significantly, but non-zero measurements were more frequently observed in caged bees. Microbial community structure differed strongly in α- and β-diversity. Caged bees showed reduced abundance of nectar-associated genera (e.g., *Apilactobacillus*) and an increase in genera that include known ethanol-producing strains, such as *Gilliamella* and *Bifidobacterium*. While we did not directly assess metabolic activity, our results suggest that captivity alters microbial communities in ways that may influence ethanol levels. This raises broader questions about how microbe-host interactions modulate host phenotypes under different environmental conditions.

**Supplementary Information:**

The online version contains supplementary material available at 10.1007/s00248-025-02627-9.

## Introduction

Ethanol is a naturally occurring substance in fermenting nectar and fruit, making it a frequent diet component for many nectar- and fruit-consuming animals [1]. In insects, ethanol often functions as an attractant and may be treated as a cue for sugar-rich food or an energy source [2]. It has been shown to attract beetles [3, 4], butterflies [5], fruit flies [6], and bees [7, 8]. In some insects, such as the Oriental hornet (*Vespa orientalis*), ethanol is metabolized with extraordinary efficiency, even at high concentrations [9]. In *Drosophila melanogaster*, ethanol-rich environments are preferred for egg-laying [6], and fruit flies can use ethanol as a self-medication agent [10].

Honeybees are no exception to this pattern. Ethanol serves as a substrate for the synthesis of ethyl oleate [11], a pheromone regulating worker maturation into foragers [12]. Laboratory studies have shown that honeybees willingly consume sugar solutions containing up to 2.5% ethanol, sometimes even preferring them to pure sucrose [8]. Honeybee workers possess alcohol dehydrogenase (ADH), an enzyme contributing to ethanol metabolism [13], and recent findings suggest that their ethanol intake increases in response to parasitic infection, indicating a potential role in infection-induced changes in behavior [14]. Even though prolonged ethanol consumption appears to exert toxic effects in honeybees [8, 15], this effect is observed only in older bees, with no detectable impact on the survival or physiological condition of relatively young workers [16]. Honeybees and possibly other insect pollinators are likely adapted to ethanol consumption [17].

One key site of ethanol processing in honeybees is the crop, a section of the foregut that functions as a nectar reservoir and site of ethyl oleate production [18]. The crop itself does not absorb the nutrients, but its contents are selectively passed to the midgut via the proventriculus [19, 20]. Passage rate depends on the substance and the physiological state of the bee. For example, glucose is transported more slowly in immobilized than in moving bees, and the emptying rate decreases with increasing solution molarity [19]. Incidentally, we detected traces of ethanol in the crop contents of caged bees that had no access to environmental food sources. This raises the possibility that microbial activity could contribute to crop ethanol accumulation in the absence of environmental ethanol exposure. In humans, a similar phenomenon is known as gut fermentation or auto-brewery syndrome [21], with such processes described in the urinary tract as bladder fermentation syndrome [22, 23]. Ethanol-producing microbes in the human gut include bacteria and fungi such as *Candida*, *Saccharomyces*, and *Klebsiella* [21, 24–27]*.*

The honeybee gut microbiota is established within days of adult emergence [28] and comprises a small set of core genera [29–32], including *Gilliamella*, *Snodgrassella*, *Lactobacillus melliventris* clade,* Bombilactobacillus*, *Bifidobacterium*, and, less abundant, *Frischella*, *Bartonella*, *Bombella*, and *Gluconobacter* [32–35]*.* These communities show large strain-level and functional diversity. All of them seem to play significant roles in digestion, immune defence, and detoxification, and show seasonal variation [29, 32, 33, 36–38], although studying the microbial impact on host physiology can be confounded by various experimental artifacts; therefore, the results need to be treated with caution [39–41]. These bacterial communities can also protect against pathogens [42–44] and modulate the effects of pesticides [45], or even produce neuroactive metabolites, such as GABA or the juvenile hormone [46, 47]. Some of these honeybee gut bacteria, such as *Gilliamella*, *Bifidobacterium*, and *Bombilactobacillus* [33, 35, 48, 49], are capable of ethanol fermentation.

The honeybee microbiota is shaped by many factors, including social transmission between bees and environmental exposure via nectar, pollen, flower surfaces, and honey [50]. The crop, though less diverse and bearing several orders of magnitude fewer bacteria than other gut regions, harbors a characteristic microbial community dominated by *Apilactobacillus kunkeei* and *Bombella apis* [18, 33, 51]. Its composition is relatively similar to that which characterizes corbicular pollen [52]. The crop microbiota participates in honey production [53] (its role in bee bread production is speculative; see the contradictory findings of [54–57]), inhibits pathogens and environmental microbes [42–44], and is transmitted between bees via trophallaxis (mouth–to–mouth food exchange) [28].

In this study, we addressed two questions: (1) How does the absence of environmental exposure affect the crop microbiota of caged bees? (2) Does this shift in microbial composition potentially contribute to crop ethanol accumulation? To address these questions, we sampled the crop contents of age-matched bees kept in natural hive conditions or laboratory cages. We assessed both microbiota composition and ethanol levels after 1 and 2 weeks. We predicted that confinement would reduce crop bacterial diversity due to the loss of environmental inputs. We further predicted that this loss of environmentally derived bacteria could open ecological space for otherwise less abundant taxa, potentially creating niches for ethanol-producing and other opportunistic species in caged bees over time.

## Methods

### Experimental Design

The experiment was conducted using queen-right colonies of *Apis mellifera carnica* Poll with naturally inseminated queens. All colonies were in good overall condition. The study was performed in three batches, each separated by a 24-h interval.

For each batch, newly emerged bees were obtained from two unrelated colonies. To achieve this, a brood frame with capped cells, free of adult bees, was collected from each colony and placed overnight in an incubator (KB53, Binder, Germany) set at 32 °C. The following morning, all newly emerged bees were marked with a colored dot on the thorax using a non-toxic paint marker and introduced into an unrelated hive, allowing them to develop naturally, including acquiring the colony microbiota and immunity.

At 7 days of age, a subset of the marked bees was collected from the hive using forceps, transferred to wooden cages, and transported to the laboratory. For each batch, four cages were established, each containing 50 individuals. Throughout the laboratory phase, bees were provided with a 40% sucrose solution and water ad libitum via gravity feeders and maintained in an incubator (KB400, Binder, Germany) at 32 °C. Sucrose and water were replenished daily, and dead individuals were removed. The remaining marked bees in the hive continued their natural development and were collected later as needed.

The first sampling was conducted 7 days later, when the bees reached 14 days of age. This time was chosen to allow the caging conditions (captivity, artificial diet, laboratory conditions) to influence the caged bees before the first sampling. Two out of the four cages per batch were removed from the incubator for sampling. Simultaneously, additional marked bees of the same age were collected from the hive and placed into two separate wooden cages (50 individuals per cage). To preserve their natural gut contents, these newly collected bees were not provided with sucrose solution or water after collection. This design resulted in two groups of same-age bees with distinct rearing environments (hereafter referred to as “bee sources”): (1) bees that had spent the previous week in the laboratory (“caged bees”) and (2) bees that had remained in the hive (“hive bees”). Crop content was sampled from living bees. Within each batch, one cage per bee source was designated for ethanol content analysis, while the other was used for microbiota metabarcoding.

The second sampling followed the same procedure after another week, when the bees reached 21 days of age. At this stage, the remaining two incubator cages from each batch were used, along with two additional cages containing newly collected, marked bees from the hive. As before, the newly collected bees were not provided with any food or water to maintain the integrity of their natural digestive tract contents.

### Ethanol Content Analysis

#### Sample Collection

Crop content was sampled individually from both bee sources (hive vs. caged bees) at two timepoints (week one vs. week two) across three batches (244 individuals in total). To obtain the crop contents, individual bees were carefully removed from their cages using forceps and gently pressed against a Styrofoam plate until they regurgitated a fluid droplet. The fluid was collected into an end-to-end microcapillary, placed in a cryotube, and immediately frozen at − 20 °C until analysis. Care was taken to avoid potential leakage from other parts of the digestive system, and visibly contaminated (opaque) samples were discarded. This non-dissection-based approach minimizes the risk of contamination from other tissues, allows direct characterization of microbes associated with the nectar-processing environment of the crop, and has been successfully applied in previous studies on other insects [58–60].

Additionally, food and water samples that the caged bees had access to in the 24 h preceding sampling, as well as freshly prepared sucrose solution and water used for feeder replenishment on the sampling day, were collected (24 samples in total). Food samples were diluted 100 × before analysis as an in-house procedure in our laboratory, covering various ethanol concentrations.

#### Ethanol assay

Ethanol levels were measured using an ethanol assay kit (K-ETOH, Neogen, USA), following the manufacturer’s protocol. Three microliters per sample was taken for analysis, with the remaining volume filled to the required 10 µl using distilled water. Samples that did not meet the minimum volume of 3 µl were either pooled or discarded if pooling was insufficient. This resulted in 127 crop samples: 91 from single bees, 35 pooled from two bees, and one pooled from three bees. Of these, 85 were from hive bees (48 in week one and 37 in week two), and 42 from caged bees (26 in week one and 16 in week two); see Online Resource 1: Table S1 for sample summary. Absorbance was measured at 340 nm using a Multiskan FC microplate reader (Thermo Scientific, USA). Each plate included a calibration curve covering the range of 0.0125–0.1 g/L ethanol. Ethanol levels were calculated using these calibration curves and corrected for the sample dilution factor. Given the 3.33 × dilution (3 µl of sample filled to the required 10 µl reaction volume with distilled water), the practical detection threshold for the assay was 0.033 g/L, based on the manufacturer’s minimum reliable detection limit of 0.01 g/L. Results below this threshold were considered below the detection limit and recorded as zero.

#### Data analysis

Statistical analysis was performed in R [61]. Ethanol levels were analyzed using a generalized linear mixed-effects model (glmmTMB, ziGamma with log link) fitted using the “glmmTMB” package [62], including bee source, timepoint, and their interaction as fixed effects, cage as a random factor, and bee source as the zero-inflation model variable. The choice of a zero-inflated model was based on the distribution of the data, where most observations were zeros. Model diagnostics were performed using the “DHARMa” package [63], confirming no significant deviations from the expected residuals distribution and uniformity and homogeneity of variances (see Online Resource 1: Fig. S1a for model formula and diagnostics).

### Microbiota Metabarcoding

#### Sample Collection

Crop content was sampled from both bee sources at two timepoints across three batches (207 individuals in total). The sampling procedure matched that used for ethanol content analysis, except that the regurgitated fluid was collected directly into sterile 0.5-mL Eppendorf tubes.

To increase the sample volume and DNA concentration, crop contents were pooled, resulting in 23 samples (from 7 to 14 bees pooled; mean ± SD = 9 ± 2.1 bees per pool). Of these, 11 samples were from the caged bees and 12 samples from the hive bees; see Online Resource 1: Table S1 for summary.

Food and water that the caged bees had access to in the 24 h preceding sampling, as well as freshly prepared sucrose solution and water used for replenishing feeders on the sampling day, were also sampled (24 samples in total, 50 µl per sample).

#### DNA Extraction and Sequencing

DNA was extracted using the Qiagen DNeasy® Blood & Tissue kit, following the manufacturer’s “Purification of Total DNA from Animal Tissues—Spin-Column Protocol” and eluted in 50 µl. One extraction blank was included. DNA concentration was measured using the Invitrogen™ Qubit™ dsDNA High-Sensitivity Assay Kit.

Library preparation and sequencing were outsourced to IGA Technology Services S.R.L. (Italy). Libraries for the V3–V4 region of the 16S rRNA gene were prepared following the Illumina 16S Metagenomic Sequencing Library Preparation protocol [64]. Universal prokaryotic primers, Pro341F and Pro805R [65], were used in the initial PCR amplification step. The subsequent amplification integrated relevant flow-cell binding domains and unique indices (Nextera XT Index Kit, FC‐131‐1001/FC‐131‐1002). Libraries were sequenced on an Aviti instrument (Element Biosciences, San Diego, USA) using a 300-bp paired-end mode. Base calling, demultiplexing, and adapter masking were performed with Bases2fastq software v.1.8 (Element Biosciences).

#### Data Preprocessing

Primer sequences were identified and trimmed using Cutadapt 4.6 [66]. Subsequent data processing and analyses were performed in R [61]. The primer-trimmed reads were denoised and merged, and chimeras were removed using the “DADA2” package [67], following the standard DADA2 pipeline tutorial [68] (see Online Resource 1: Table S2 for the number of reads).

#### Classification and Decontamination

Taxonomy was assigned to the amplicon sequence variants (ASVs) using the IdTaxa function [69] from the “DECIPHER” package [70], with the BEExact reference database (BEEx_v2023.01.30___idtaxa_v3v4.RData), which is designed for 16S rRNA-based studies of bee-associated microbiota [71]. ASVs not reaching at least 10 × the relative abundance of the blank sample in any of the experimental samples were classified as contaminants and removed [72].

#### Community Composition Analyses

Further analyses were performed and visualized using the “phyloseq” package [73] with additional functions from “speedyseq” [74] and “microbiome” [75]. Visualizations were done with additional usage of “BioVenn” [76, 77], “ggplot2” [78], “palletter” [79], “RColorBrewer” [80], “ggpubr” [81], and “cowplot” [82] packages. In data processing and analysis, packages “Biostrings” [83], “readxl” [84], “writexl” [85], “dplyr” [86], and “tidyverse” [87] were used.

Two α-diversity measures: the number of observed variants (ASV richness) and Shannon index were calculated using the estimate_richness function and visualized with the plot_richness function from “phyloseq.” Differences in α-diversity (ASV richness and Shannon index) between hive bees and caged bees, as well as between sampling timepoints (week one and week two) and their interaction, were tested using a generalized linear mixed-effects model (generalized Poisson distribution with log link for ASV richness as these represent count data; lognormal distribution for the Shannon index) fitted using the “glmmTMB” package [62]. DNA concentration in the sample and the number of individual samples in the pool were used as additional factors; cage was used as a random factor. Model performance was assessed using the “DHARMa” package [63], confirming no significant deviations from the expected residuals distribution and uniformity and homogeneity of variances (see Online Resource 1: Fig. S1b,c for α-diversity model formulas and diagnostics).

To test whether bacterial communities in bee samples and feeders differ, constrained correspondence analysis (CCA) with the “vegan” package [88] was performed with sample type (bee sample vs. feeder) as a variable. Further, β-diversity among bee samples was also analyzed using CCA. The model included bee source (hive vs. cage), timepoint (week one vs. week two), their interaction, and cage as predictors. A permutation test for the CCA model was performed using the anova.cca function from “vegan” using 999 permutations. To test differences in within-group variability (beta-dispersion) across bee sources (hive bees vs. caged bees), we used the betadisper function from the “vegan” package. This function calculates the distance of each sample to the spatial median of its group based on a Bray–Curtis dissimilarity matrix. We enabled the bias.adjust = TRUE argument to correct for small sample sizes. Group-level differences in dispersion were then tested using ANOVA on the resulting object.

To visualize the similarities between the microbial composition of bees and food/water samples, as well as between bee sources, non-metric multidimensional scaling (NMDS) plots based on Bray–Curtis dissimilarities were prepared using the plot_ordination function from the package “phyloseq.” Additionally, we calculated the distance of each sample to the spatial median of each group (hive bees, caged bees, food, water) with the betadisper function.

To visualize and perform tests on community composition at specific taxonomic levels, datasets were aggregated to the chosen taxonomic level using the aggregate_rare function, applying a detection cutoff of 300 reads per sample and a prevalence threshold of 20% of samples. Variants below these thresholds were categorized as “Other”. The microbial composition for the whole dataset was visualized at the family level and for the bee samples—additionally at the genus level. Heatmaps were generated on the bee samples data aggregated to the genus level.

Differential abundance of bacterial genera in the bee samples was analyzed using “DESeq2” [89], with Benjamini–Hochberg correction for multiple comparisons [90], using the dataset aggregated to genus level as described above. All 20 genera resulting from the aggregation were included in the analysis. Genus-level abundances were considered statistically significant at *p* < 0.05. Similarly, the differential abundance of ASVs was analyzed using “DESeq2” on count data. On the ASV level, the first two comparisons were conducted on ASV log-twofold-change between hive bee crop vs. food samples and between hive bee crop vs. water samples, to identify variants significantly more abundant in the provided feeders than in the natural honeybee crop microbiota. These variants were marked as “bacteria enriched in food” and “bacteria enriched in water,” respectively, in the ultimate ASVs comparison between hive bees vs. caged bees crop samples. In all the ASV differential abundance analyses, a significance threshold of *p* < 0.01 was used to avoid discovering too many false-positive differences in ASV abundance.

## Results

### Ethanol Content

**Fig. 1:**
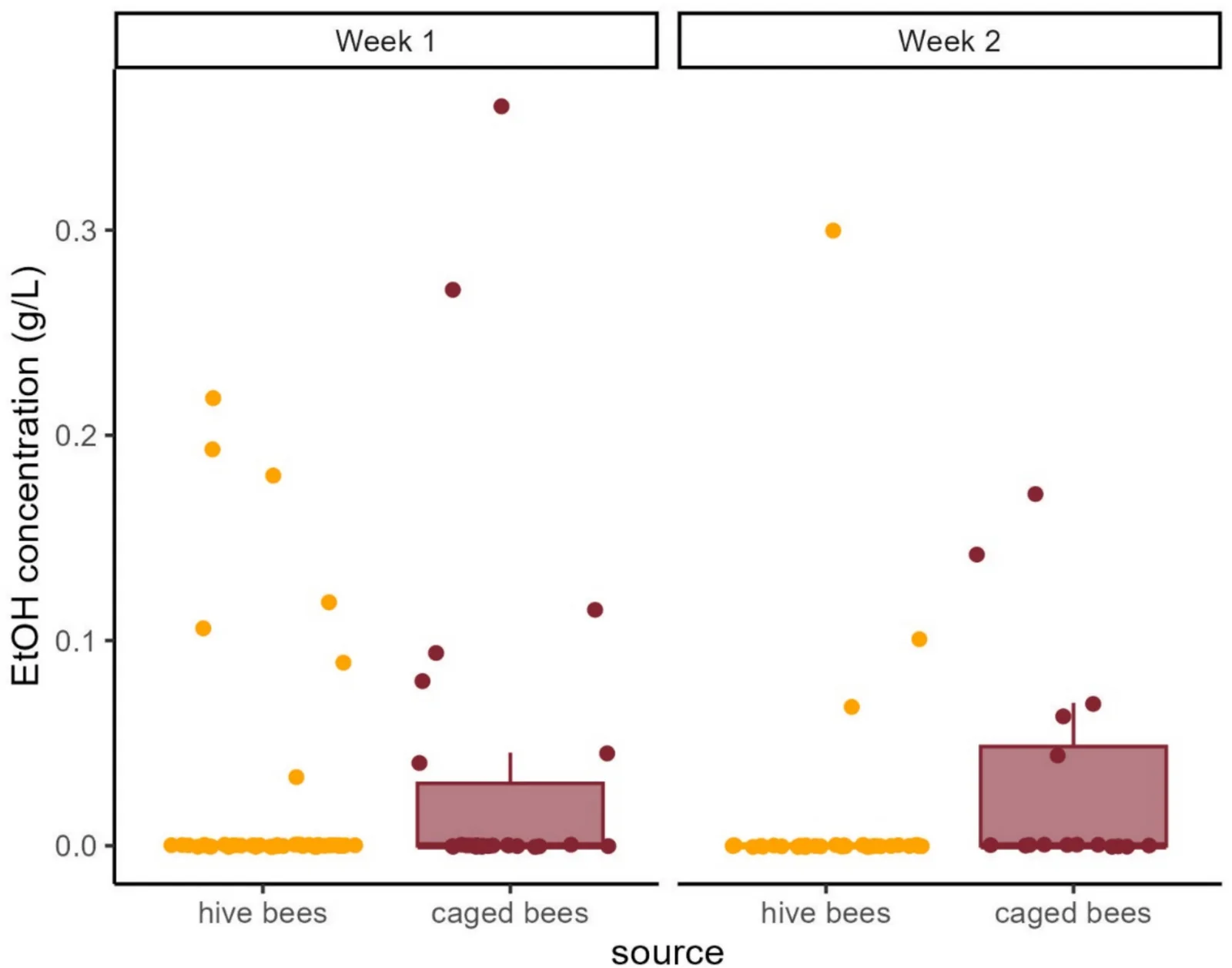
Ethanol concentrations in bee crop samples across sources and weeks. Boxplots show ethanol levels for hive and caged bees at two timepoints (week one and week two). Each dot represents an individual sample. Boxes indicate medians and interquartile ranges (IQR), with whiskers extending to 1.5 × IQR

Ethanol levels in the crop samples were consistently low across bee sources and timepoints, ranging from 0 to 0.35 g/L, with all group medians close to 0 (Fig. 1, Table 1). No ethanol was detected in water or food samples. There were no significant differences in ethanol levels between bee sources (χ^2^ = 0.2013, df = 1, *p* = 0.65), between weeks (χ^2^ = 0.3451, df = 1, *p* = 0.56), or in their interaction (χ^2^ = 0.9445, df = 1, *p* = 0.33). The estimated variance for the random effect was negligible, on the order of 10^−10^, indicating minimal contribution of cage to model variance. However, the zero-inflation component of the model indicated that caged bees had significantly more samples with detectable ethanol levels compared to hive bees (estimate ± SE = −1.099 ± 0.480, z = −2.291, *p* = 0.022).

**Table 1:** Results of the zero-inflated linear model testing ethanol levels in bee crop samples. The conditional model estimates effects on log-transformed ethanol concentration, using hive bees at week one as the reference. The zero-inflation model estimates the likelihood of samples having undetectable ethanol. Only the zero-inflation component showed a significant effect, with caged bees more likely to contain detectable ethanol

Model	Estimate	Standard error	z value	Pr(>|z|)
Conditional model				
Intercept	-2.00812	0.23125	-8.684	< 2e-16
Caged bees	0.06932	0.32704	0.212	0.832
Week two	0.15239	0.42221	0.361	0.718
Caged bees × Week two	-0.53814	0.55372	-0.972	0.331
Zero-inflation model				
Intercept	2.0149	0.3367	5.985	2.16e-09
Caged bees	-1.0986	0.4796	-2.291	0.022

### Microbiota

Sequencing of the libraries resulted in 22,264,863 read pairs, on average 473,720 per sample. Among them, after primer-trimming, we identified 11,751 ASVs, out of which 7255 were classified as bacterial ASVs. Among the bacterial ASVs, 151 were classified as contaminants based on their relative abundance in the blank sample. The distribution of contaminants across samples is shown in Online Resource 1: Fig. S2. The most common contaminants in the extraction blank belonged to the families: *Rhizobiaceae*, *Sphingomonadaceae*, and *Pseudomonadaceae* (Online Resource 1: Fig. S3), which is consistent with previous reports of common ultrapure water and next-generation sequencing contaminants [91, 92]. The final dataset included 7104 ASVs, represented by 1–1,826,393 read counts per variant.

#### α-Diversity

**Fig. 2:**
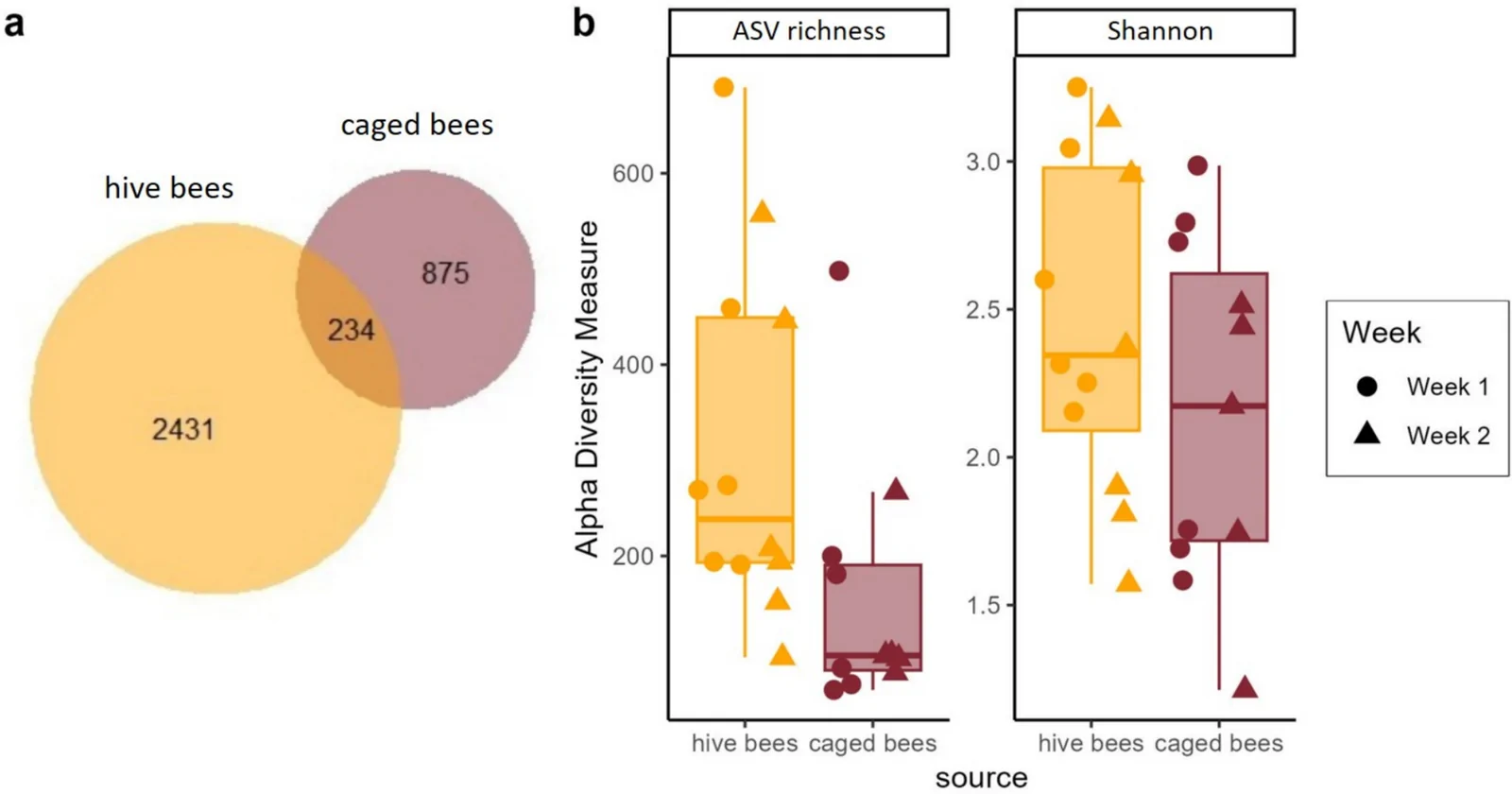
Alpha diversity of bacterial communities in hive and caged bees.** a** Venn diagram showing the number of ASVs shared and unique to hive and caged bees. **b** ASV richness (left) and Shannon diversity index (right) plotted by bee source. Caged bees had significantly lower ASV richness, while Shannon diversity did not differ significantly

A total of 875 ASVs were identified in caged bees and 2431 ASVs in hive bees, with 234 ASVs (6.6%) shared between the two sources (Fig. 2a). ASV richness was significantly lower in caged bees than in hive bees (*F* = 9.174, *p* = 0.0076; Fig. 2b). However, neither timepoint (*F* = 0.924, *p* = 0.35), nor the interaction between bee source and timepoint (*F* = 0.014, *p* = 0.91) was significant. DNA concentration in the sample (*F* = 1.338, *p* = 0.26) as well as the number of samples in a pool (*F* = 3.271, *p* = 0.088) also had no significant effect on ASV richness. The random effect of cage explained little variance (variance ± SD: 0.017 ± 0.129).

The Shannon index did not differ significantly between bee sources (*F* = 1.485, *p* = 0.24), timepoints (*F* = 1.258, *p* = 0.27), or their interaction (*F* = 0.1371, *p* = 0.72) (Fig. 2b). Neither DNA concentration in the sample (*F* = 1.064, p = 0.32) nor the number of samples in a pool (*F* = 0.047, *p* = 0.83) had a significant effect on the Shannon index. The random effect of cage explained little variance (variance ± SD: 0.018 ± 0.136).

#### β-Diversity

**Fig. 3:**
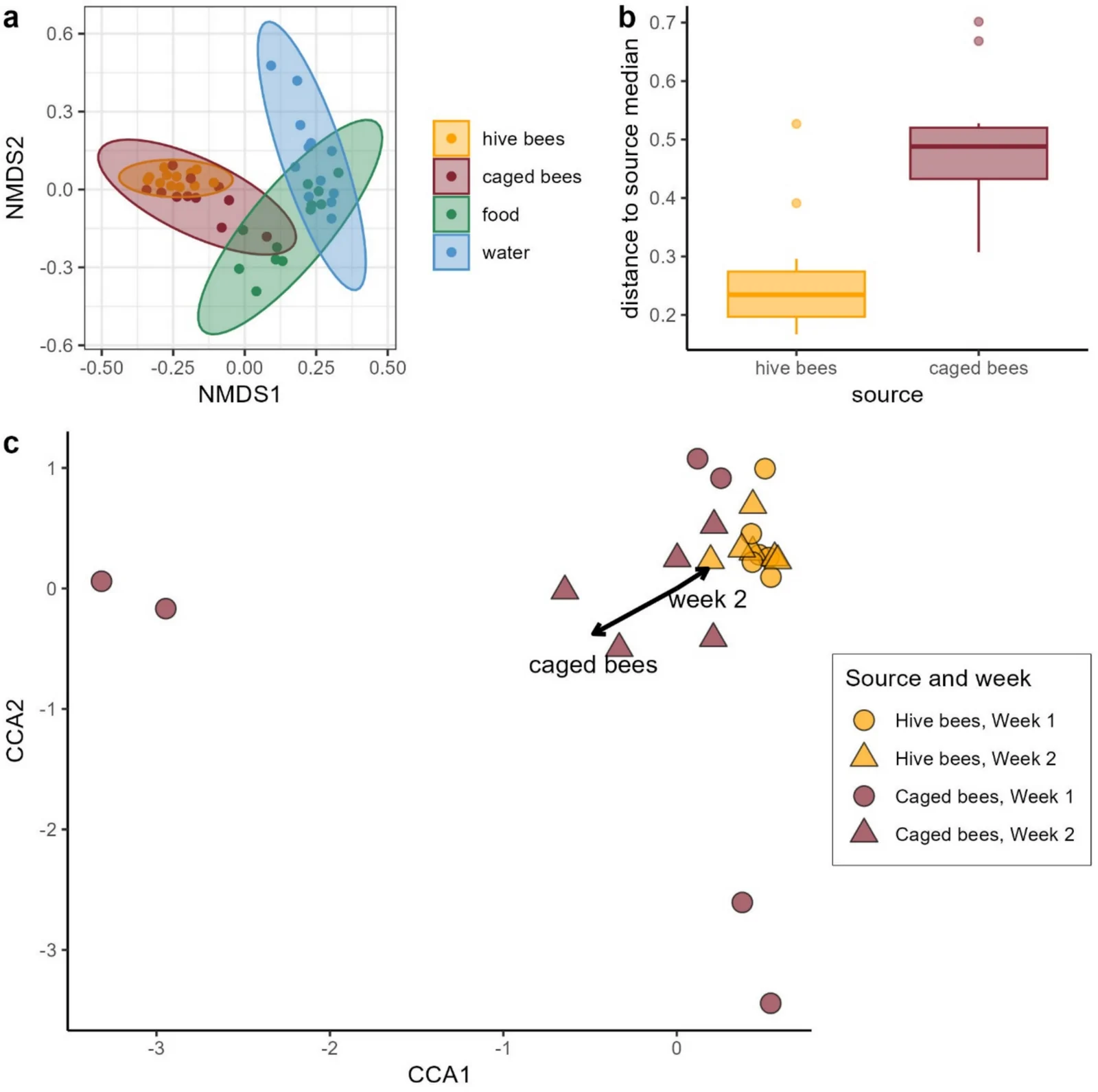
Beta diversity of crop microbiota across bee sources and feeders.** a** NMDS ordination of Bray–Curtis dissimilarities shows that bee microbiota cluster separately from food and water samples, with hive and caged bees broadly overlapping. Stress = 0.15. **b** Boxplots showing within-group beta-dispersion; caged bees had significantly greater dispersion than hive bees (*p* < 0.001). **c** CCA plot showing effects of bee source, timepoint, and cage on microbial community composition. CCA1 and CCA2 explain 61% and 41% of the constrained variance, respectively. Cage-specific axes are omitted for clarity

Bacterial communities in samples from bees were different from those from feeders (CCA ANOVA: χ^2^ = 0.75, *p* = 0.001, Fig. 3a). Bray–Curtis dissimilarity NMDS clustering revealed that food and water samples form distinct clusters, separated from bee samples (Fig. 3a), indicating that the microbial communities of bees and their feeders were compositionally distinct. Bee microbial communities from both sources (hive and caged) largely overlapped (Fig. 3a), though hive bees formed a more condensed cluster while caged bees showed greater dispersion (ANOVA test result for the betadispersion model: *F* = 25.874, *p* < 0.001; Fig. 3b). Two caged bee samples clustered closely to hive bees and one overlapped with food samples (Fig. 3a, Online Resource 1: Table S3).

Constrained correspondence analysis (CCA) identified significant effects of the bee source, timepoint, and cage on microbial community composition (Table 2, Fig. 3c, Online Resource 1: Fig. S4). There was an evident separation between weeks in the caged bees (Fig. 3c). In total, the model explained 69% of the observed variance (Online Resource 1: Table S4).

**Table 2:** Effects of bee source, timepoint, and cage on microbial community composition (CCA ANOVA). All predictors had significant effects on beta diversity. Degrees of freedom (Df), Chi-squared statistic (χ^2^), *F*-value, and associated *p*-values are shown

	**Df**	**χ**^**2**^	**F**	**Pr(> F)**
Bee source	1	0.33679	3.6782	0.001
Timepoint	1	0.18720	2.0445	0.001
Cage	9	1.73693	2.1077	0.001
Residual	11	1.00722

#### Bacterial Communities

**Fig. 4:**
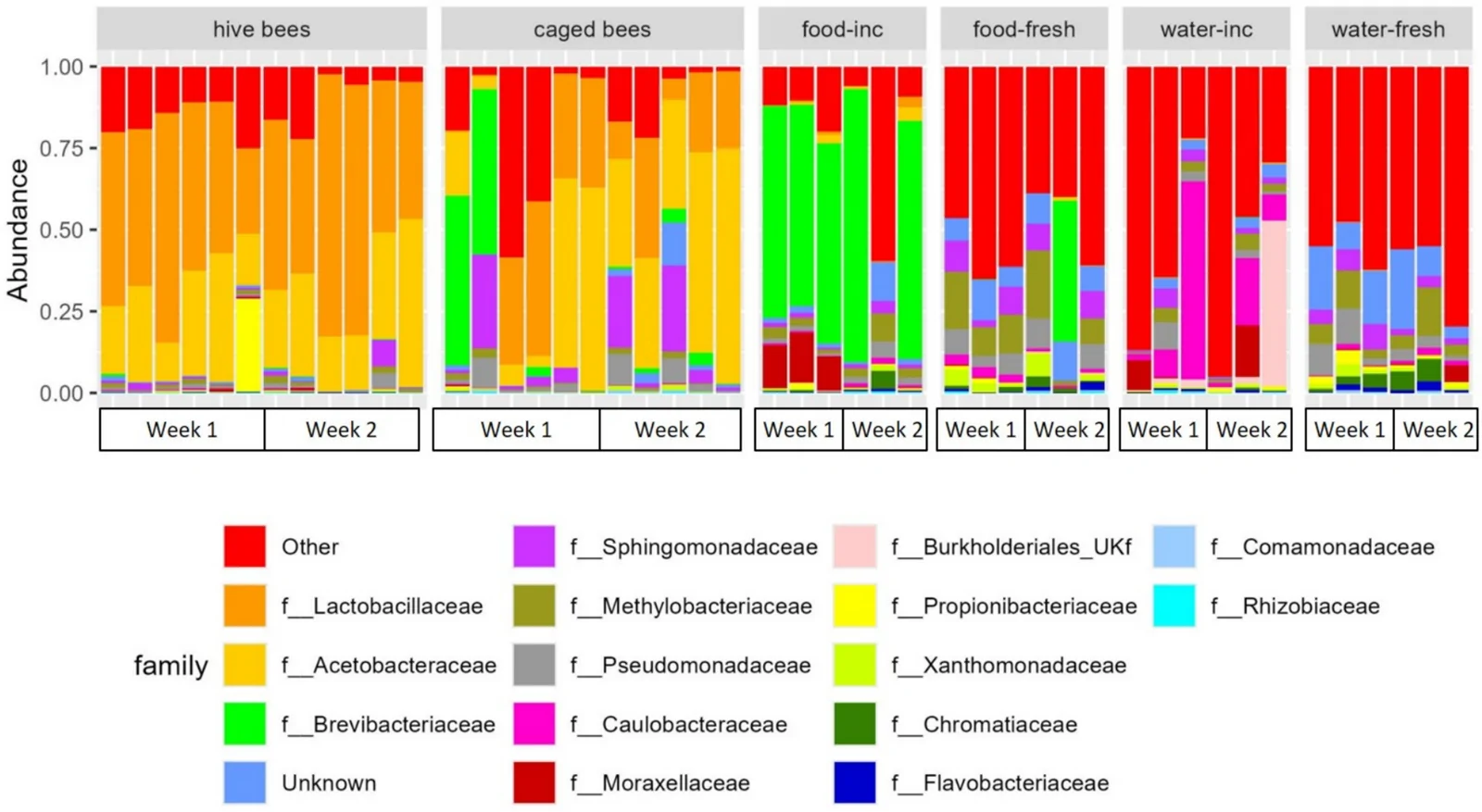
Family-level composition of bacterial communities in bee, food, and water samples. Bee samples were dominated by *Lactobacillaceae* and *Acetobacteraceae*, which were nearly absent in feeders. Food and water samples showed more diverse profiles, with *Brevibacteriaceae* dominating incubated food. Labels: “inc” = incubated 24 h, “fresh” = freshly prepared

At the family level, bee samples were mostly composed of *Lactobacillaceae* and *Acetobacteraceae*, which were almost absent from food and water samples (Fig. 4). Food and water samples exhibited greater diversity, with incubator food samples dominated by *Brevibacteriaceae*, which were also present in high abundance in two caged bee samples from week one, but were less abundant or absent in other caged bee or hive bee samples from week two (Fig. 4).

**Fig. 5:**
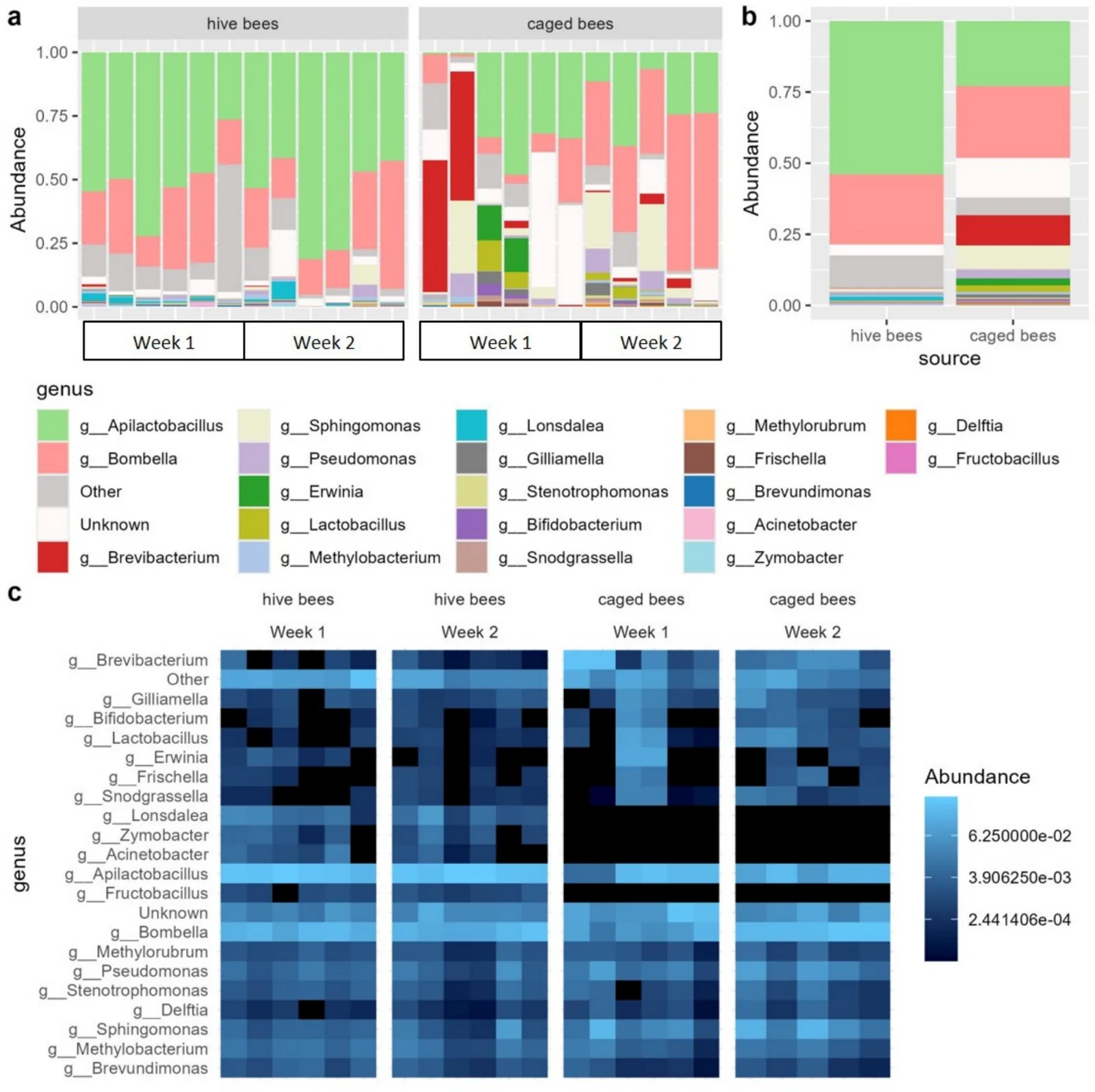
Genus-level composition of bacterial communities in bee crop samples.** a** Stacked bar plots for individual hive (left) and caged (right) bee samples. **b** Mean relative abundance per bee source. **c** Heatmaps showing genus-level composition per sample, separated by bee source and week. Hive bees showed stable community structure, while caged bees were more heterogeneous, particularly in week one

**Fig. 6:**
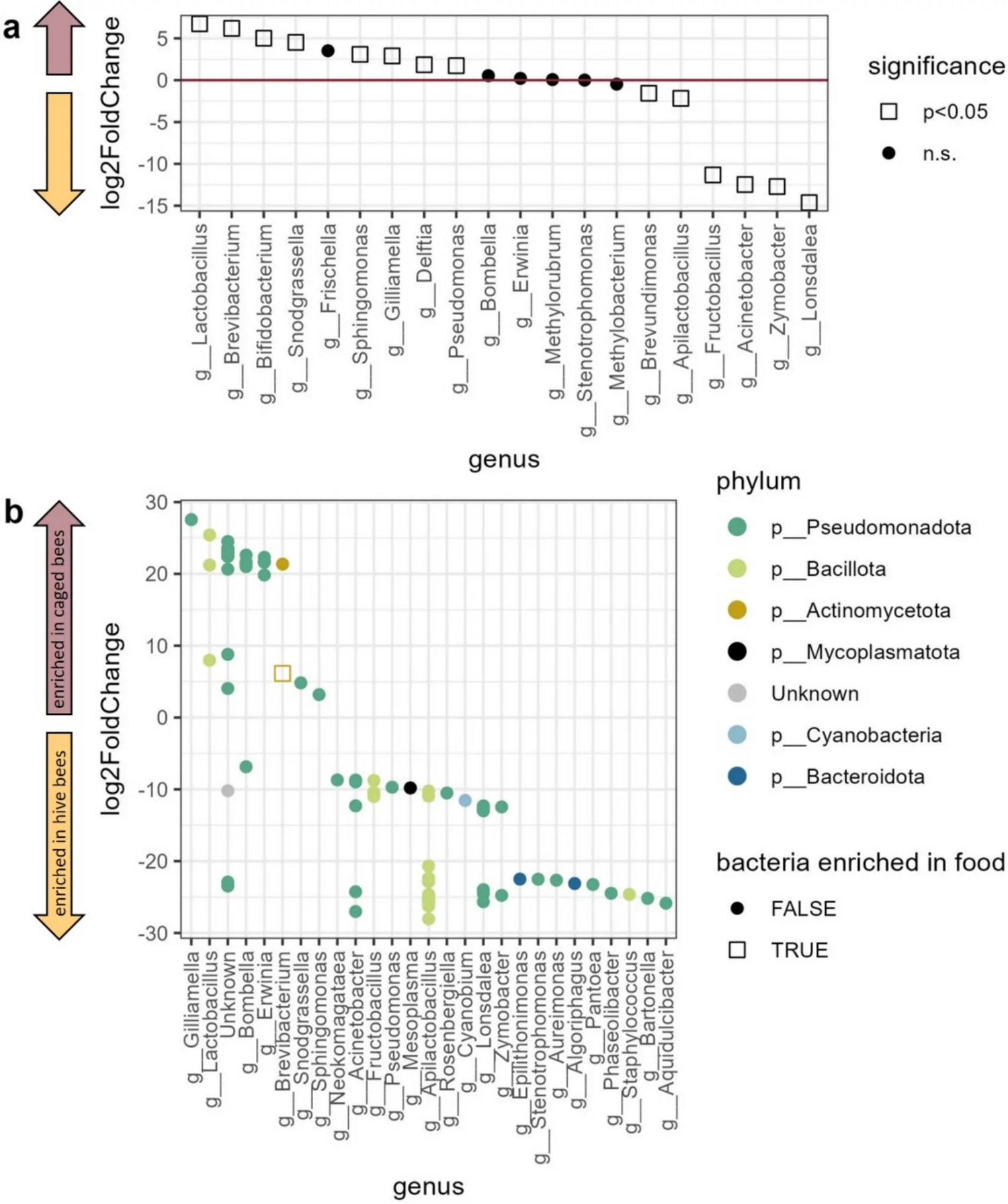
Differential abundance analysis of bacterial taxa between hive and caged bees.** a** Log2 fold changes in genus-level abundance from DESeq2. Genera enriched in caged bees are shown above the zero line (brown upward arrows) and those enriched in hive bees, below (golden downward arrows). Significance (FDR < 0.05) is indicated by squares; circles indicate non-significant genera. **b** Log2 fold changes for individual ASVs (FDR < 0.01). Positive values indicate ASVs enriched in caged bees (brown upward arrows) and negative values in hive bees (golden downwards arrows)

The most abundant genera in both hive and caged bee samples were *Apilactobacillus* and *Bombella* (Fig. 5a, b). However, hive bees’ crop samples were characterized by a greater number of relatively abundant genera (Fig. 5c). The overall composition in hive bees was relatively similar between weeks, while the caged bees exhibited higher dissimilarity in week one, becoming less heterogeneous in week two (Fig. 5a, c). On average, *Apilactobacillus* and *Bombella* together constituted over 75% of the crop bacterial community in hive bees, while in caged bees, these two dominant genera accounted for less than 50% of the crop microbiota (Fig. 5b). This difference was primarily driven by the higher abundance of *Apilactobacillus* in hive bees, while *Bombella* levels did not differ between bee sources (Figs. 5b and 6a). In contrast, caged bee samples were enriched in the next two common genera, *Sphingomonas* and *Pseudomonas* (Fig. 6a), and six less common genera: *Lactobacillus*, *Brevibacterium*,* Bifidobacterium*, *Snodgrassella*, *Gilliamella*, and *Delftia* (Fig. 6a). Genera enriched in the hive bees included, apart from the most common *Apilactobacillus,* also *Lonsdalea*, *Zymobacter*, *Acinetobacter*, *Fructobacillus*, and *Brevundimonas* (Fig. 6a)*.* In total, out of the 20 compared genera, six were significantly less abundant in caged bees, and eight were enriched in the caged bees (Fig. 6a).

In the differential abundance analysis (DESeq2) on the ASV level, the comparison between hive bees and food samples identified 27 variants significantly more abundant in food than in the natural honeybee crop microbiota. These variants were classified as “bacteria enriched in food” (Online Resource 1: Fig. S5, top panel). Analogously, 52 ASVs were identified as “bacteria enriched in water” based on the comparison with water samples (Online Resource 1: Fig. S5, bottom panel). In the primary comparison between hive and caged bee samples, 57 ASVs were significantly more abundant in hive bees, while 34 ASVs were significantly more abundant in caged bees (*p *< 0.01). Of the ASVs more abundant in caged bees, only one variant, belonging to *Brevibacterium,* was classified as “bacteria enriched in food,” while none was classified as “bacteria enriched in water” (Fig. 6b). Among the core microbiota in the bee samples, two genera, *Apilactobacillus* and *Bombella*, showed contrasting patterns. Namely, *Apilactobacillus* had multiple variants overrepresented in hive bees and no ASVs characteristic for caged bees, while *Bombella* included several variants overrepresented in caged bees and only one variant that was significantly more abundant in hive bees. Other variants significantly more abundant in caged bees than in hive bees were identified as representatives of a few other genera: *Gilliamella, Lactobacillus, Erwinia, Brevibacterium*, *Snodgrassella*, and *Sphingomonas*.

## Discussion

Our experiment showed that honeybees kept in laboratory conditions exhibit shifts in their crop microbiota compared to hive-reared bees. This effect was expected, as the crop microbiota is highly affected by the environmental supply of bacteria [50]. However, the crop microbiota of caged bees remained distinct from the microbial composition of the food and water they received, but rather represented a reduced and more variable subset of the hive bee microbiota. This was observed as decreased ASV richness and higher β-diversity in caged bees compared to hive bees.

Although the regurgitated fluid may not contain all the bacteria inhabiting the crop, due to the biofilm formation (as mentioned by [93]), major crop microbiota components captured in our experimental bees were in line with the literature. The most abundant bacterial genus in both groups, *Apilactobacillus*, was notably less abundant in caged bees, consistent with its known environmental origin (hive surfaces and flowers). In contrast, *Bombella*, another core crop taxon, maintained stable abundance across groups. Other genera showed shifts in both directions. The increased relative abundance of *Gilliamella* and *Bifidobacterium* in caged bees raises the question of whether some taxa may contribute to endogenous ethanol accumulation, although this remains to be directly tested.

While ethanol content in the crop samples remained low (mean ~ 0.01 g/L) and did not significantly differ in magnitude between groups, it was detected more frequently in caged bees. Some ethanol-positive samples were also observed in hive bees, consistent with the likelihood that bees encounter low levels of ethanol in the environment or within the hive [1, 17]. The detected levels were approximately two orders of magnitude higher than in hemolymph of bees reared under similar laboratory conditions [14] and in bees fed ethanol-spiked sucrose [94]. These levels are consistent with low-level ethanol presence in the crop and raise the possibility that fermentation may occur independently of environmental ethanol intake, although alternative explanations cannot be ruled out.

Despite a shared composition (Fig. 3a), the crop communities of caged bees were more heterogeneous (Fig. 3b), indicating potentially unstable microbial communities. This heterogeneity in the caged bees group can be in part driven by differences between individual cages (see [40]). The instability may reflect the unnatural, microbially impoverished incubator environment, which lacks the diverse microbial inputs typically encountered by foraging bees [28]. Hive microbiota is, on one hand, dependent on its surrounding environment, and, on the other hand, serves as a microbial reservoir of high importance for colony health [95].

The low prevalence of bacteria dominant in caged bee samples within their food and water sources suggests minimal microbial transmission. In contrast, the temporary spike in *Brevibacterium* abundance observed in some caged bees in week one likely resulted from contamination with incubator food, where this genus was abundant (Fig. 5). This transient contamination appears to have destabilized the microbiota of some caged bees but was not observed in week two, suggesting resilience. It also may have contributed to the greater differences observed between samples originating from caged bees, mainly driven by the samples from week one (Fig. 5), especially given the significant effect of the cage detected in CCA.

Reduced ASV richness in caged bees compared to hive bees aligns with expectations, given the lack of environmental microbial inputs and the absence of hive-associated microbial exchange. This effect is consistent with previous findings that 22.5% of honeybee-associated bacterial variants, primarily those found on the body surface, are also present in flowers and are typically acquired indirectly through honey stored in the hive [50]. The two most abundant genera in both hive and caged bee samples, *Apilactobacillus* and *Bombella,* align with known crop microbiota components [18, 33–35, 43, 44, 96]. The most striking difference between hive and caged bees was observed in the reduced abundance of *Apilactobacillus* in caged bees (Figs. 5 and 6a). This fructophilic genus, a known component of corbicular pollen [52] and a key player in floral microbiomes [35, 97], is likely acquired primarily from environmental sources; it is also a component of hive microbiota [44]. The absence of environmental sources in caged bees, as well as no access to hive material, may account for their reduced *Apilactobacillus* levels.

Interestingly, *Bombella* abundance did not differ significantly between hive and caged bees. This stability may reflect a greater tolerance for laboratory diet caused by its preference for glucose as a carbon source [44], making this genus less sensitive to environmental isolation. In contrast, the genus *Gilliamella*, typically transmitted via social contact [49], especially with the fecal material [28], was more abundant in the crops of caged bees. This result is surprising, as previous studies have reported reduced *Gilliamella* abundance in the ileum of bees without hive contact [98], suggesting that the relationship between gut compartment and microbiota composition is complex and potentially environment-dependent.

*Lactobacillus* and *Bifidobacterium*, both of which are commonly found in the honeybee crop [28, 43, 53], were more abundant in caged bees than in hive bees. This increase might be plausibly explained by the stability and carbohydrate-rich nature of the laboratory diet, which favors lactic acid bacteria that can metabolize sucrose [99–101]. Similarly, *Acinetobacter*, one of the most abundant bacterial genera found in floral nectar [102], was rare in caged bees, likely reflecting the loss of regular environmental input. *Fructobacillus*, a fructose-specialist commonly found in flower nectar [103], was completely absent from the caged bees in this study. These observations are consistent with the idea that the loss of nectar-associated microbes in caged bees could create ecological opportunities for other taxa [43].

The hive bee crop microbiota was enriched in variants belonging to genera commonly associated with floral nectar, including *Acinetobacter*,* Fructobacillus*,* Pseudomonas*,* Rosenbergiella*, and *Pantoea* [102, 104]. Interestingly, one genus, *Bombella*, showed some strain-specificity, with separate variants enriched in caged and hive bees. This finding is in line with the generally reported high functional diversity within genera of bacterial microbionts [29–32, 105].

The presence or relative enrichment of taxa known to be capable of ethanol fermentation, such as *Gilliamella* and *Bifidobacterium*, although suggestive [33, 48, 49], does not confirm metabolic activity in vivo. Moreover, caging profoundly alters bee physiology and behavior, introducing multiple confounding factors [40, 41]. In such constrained conditions, they lack social cues and cannot forage outside, which increases their stress levels [106] and decreases immune responses [107]. Such changes may interfere with ethanol metabolism or clearance, potentially leading to low-level accumulation even without microbial fermentation. A more nuanced interpretation is therefore warranted: laboratory confinement may modulate both microbiota composition and host physiology, together enabling ethanol accumulation in the crop. To disentangle these mechanisms, future work should employ functionally explicit methods, such as ex vivo fermentation assays under crop-like conditions, metatranscriptomics or metabolomics to identify active fermentation pathways, or experiments with sterile bees and microbiota manipulations, e.g., antibiotics or gnotobiotic setups [29, 36, 37].

Our results are in line with recent findings [96] that the crop microbiota is shaped by the environmental sources only to some extent, much less than the mouth microbiota, and some “core taxa” are present in the crop regardless of their external supply. This allows us to predict that some key microbiota functions are preserved even in the disturbed artificial environment. For example, abundances of several bacterial genera, known for their role in honeybee health [42, 43, 50], were not negatively affected (*Bombella*), or even increased, like *Lactobacillus*,* Bifidobacterium*, and* Pseudomonas*. Also, bacteria involved in nutritional functions and carbohydrate utilization, such as *Gilliamella* and *Snodgrassella*, which engage in cross-feeding [37, 108], were highly abundant in caged bees. However, it should be noted that, as relative abundances were analyzed, it cannot be determined if bacteria were less or more abundant in terms of absolute values between bee sources.

## Conclusions

Our findings are consistent with the hypothesis that laboratory confinement reduces crop microbiota richness in honeybees and is associated with increased relative abundance of opportunistic and potentially ethanol-producing taxa such as *Gilliamella* and *Bifidobacterium*. At the same time, some core components, like *Bombella*, remained stable, suggesting the resilience of key symbionts. However, the precise relationship between these microbial shifts and ethanol production remains to be fully explored. Our results can be viewed as a preliminary insight into the topic. Future studies should also investigate how ethanol exposure itself may shape the microbiota and should integrate both bacterial and fungal communities, especially given the influential roles of fungi in crop microbiota [109] (but see [110]). Assessing the functional consequences of these shifts, including potential impacts on host physiology, behavior, and health, is essential for understanding how microbial symbionts contribute to honeybee phenotypic plasticity.

## Supplementary Information

Below is the link to the electronic supplementary material.

ESM (PDF. 1.47 MB)

## Data Availability

The datasets including the complete list of samples used for ethanol measurements and metabarcoding, including assignment to experimental groups, batches and pooling information, are available in the RepOD repository [https://repod.icm.edu.pl/; direct link to datasets: 10.18150/QTRG45]. The nucleotide sequence data reported are available in the Sequence Reads Archive [http://www.ncbi.nlm.nih.gov/sra] under the project number PRJNA1271430.
