# Supplementary material for: Do Shifts in Honeybee Crop Microbiota Enable Ethanol Accumulation? A Comparative Analysis of Caged and Foraging Bees
Source: Microb Ecol. 2025 Dec 4;88(1):137. doi: 10.1007/s00248-025-02627-9 (PMC12680868; doi:10.1007/s00248-025-02627-9)
Supplement: Supplementary file 1 — (PDF. 1.47 MB) [file 248_2025_2627_MOESM1_ESM.pdf]

Supplementary material

Do shifts in honeybee crop microbiota enable ethanol accumulation? A comparative analysis of caged and foraging bees

Microbial Ecology

Weronika Antoł[1], Bartłomiej Surmacz[1], Monika Ostap-Chec[1], Daniel Stec[1], Krzysztof Miler[1]

[1] Institute of Systematics and Evolution of Animals of the Polish Academy of Sciences, Kraków, Poland

Corresponding author: Weronika Antoł[1], e-mail: antol@isez.pan.krakow.pl

Table S1. Number of pooled samples per category for ethanol level measurement and microbiota analysis, divided by weeks. In parentheses, the numbers are split into particular cages. In case of bee crop samples, the bee crop content of several individuals was pooled to increase sample volume. In the ethanol assay, 127 pooled crop samples were analyzed: 91 from single bees, 35 pooled from two bees, and one pooled from three bees. In the microbiota analysis, 23 pooled samples were analyzed, each from 7-14 bees pooled: mean ± SD = 9 ± 2.1 bees per pool.

	**Ethanol**		**Microbiota**	
**Sample source**	Week 1	Week 2	Week 1	Week 2
**Bee crop**				
caged bees	26 (18 + 8)	16 (6 + 3 + 7)	6 (2 + 2 + 2)	5 (2 + 2 + 1)
hive bees	48 (24 + 17 + 7)	37 (17 + 8 + 12)	6 (2 + 2 + 2)	6 (2 + 2 + 2)
**Food**				
fresh	6 (2 + 2 + 2)	6 (2 + 2 + 2)	3 (1 + 1 + 1)	3 (1 + 1 + 1)
incubator	6 (2 + 2 + 2)	6 (2 + 2 + 2)	3 (1 + 1 + 1)	3 (1 + 1 + 1)
**Water**				
fresh	6 (2 + 2 + 2)	6 (2 + 2 + 2)	3 (1 + 1 + 1)	3 (1 + 1 + 1)
incubator	6 (2 + 2 + 2)	6 (2 + 2 + 2)	3 (1 + 1 + 1)	3 (1 + 1 + 1)

a

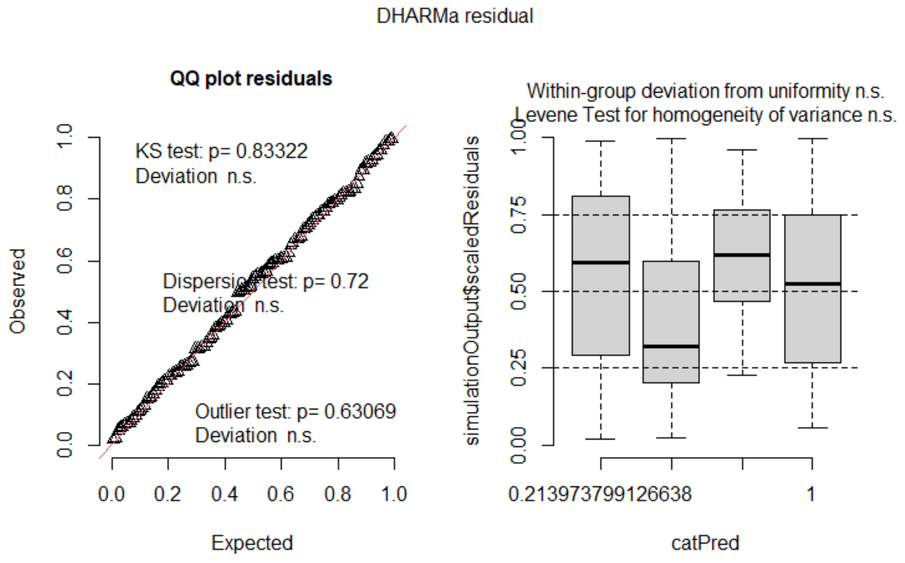

b

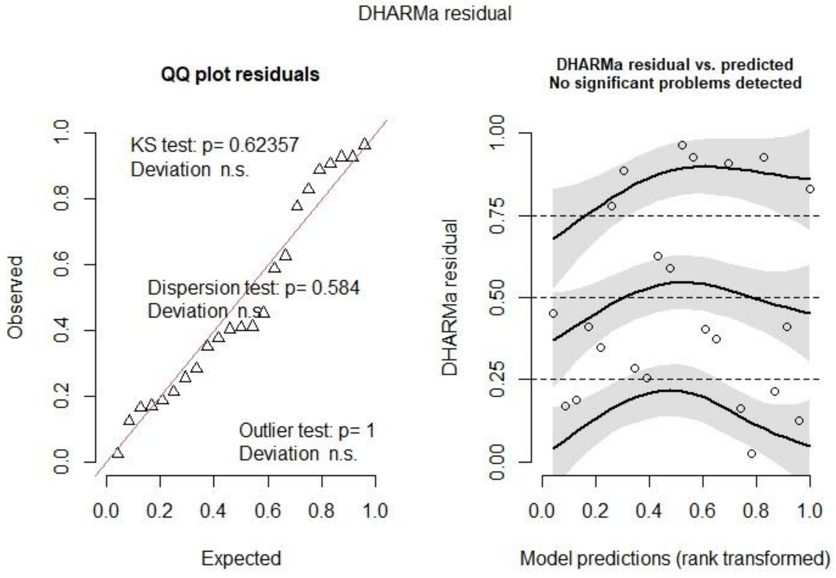

c

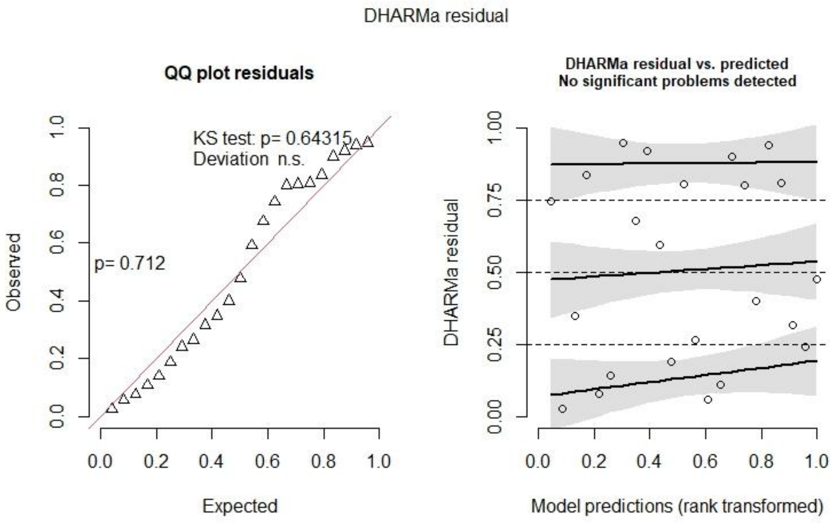

Fig. S1. DHARMa diagnostic plots for the glmmTMB models. The qqplot plot (left) shows no significant deviation from the expected distribution. The residuals plot (right) shows no significant deviation from the assumptions of uniformity and homogeneity of variances **(a) ethanol levels analysis.** Model formula: glmmTMB(EtOH ~ bee source*timepoint + (1|cage), ziformula = ~ bee source, family = ziGamma(link="log")). **(b) ASV richness.** Model formula: glmmTMB(ASV richness ~ bee source*timepoint + DNA concentration in the extract + number of samples in pool + (1|cage), family = genpois. (c) **Shannon index.** Model formula: glmmTMB(Shannon index ~ bee source*timepoint + DNA concentration in the extract + number of samples in pool + (1|cage), family = lognormal.

Table S2. Number of reads for each sample at several stages of data preprocessing: original raw reads (INPUT), and after: quality filtering and trimming (FILTER), denoising (DENOISED_F and DENOISED_R for forward and reversed reads, respectively), merging the F and R reads (MERGED), removing chimeras (NONCHIM). The last column (%) shows the percentage of original reads passed to further analysis.

	INPUT	FILTERED	DENOISED_F	DENOISED_R	MERGED	NONCHIM	%
**M1-R2-T1-C-1-16S**	540343	446835	444306	444492	392587	386086	71%
**M1-R2-T1-C-2-16S**	434538	354571	353479	353375	337318	329287	76%
**M1-R2-T1-U-1-16S**	718100	616470	613101	611679	460375	446688	62%
**M1-R2-T1-U-2-16S**	278047	225292	224157	224002	168204	164261	59%
**M1-R2-T1-P-C-16S**	312155	248430	247666	247499	239338	238043	76%
**M1-R2-T1-P-F-16S**	568769	482788	481417	482187	404857	401797	71%
**M1-R2-T1-W-C-16S**	592663	493253	491085	491555	454457	446904	75%
**M1-R2-T1-W-F-16S**	402936	332853	326497	326360	206103	186909	46%
**M1-R2-T2-C-1-16S**	246322	215608	214811	214970	175048	166742	68%
**M1-R2-T2-C-2-16S**	1326357	1097069	1093191	1093653	832342	798789	60%
**M1-R2-T2-U-1-16S**	657790	538773	535814	535499	398665	389369	59%
**M1-R2-T2-U-2-16S**	614716	485976	483158	483070	373299	363301	59%
**M1-R2-T2-P-C-16S**	320494	233639	231176	233198	226439	225646	70%
**M1-R2-T2-P-F-16S**	463522	379996	378239	379138	335621	327359	71%
**M1-R2-T2-W-C-16S**	445788	345125	341865	342896	233611	232285	52%
**M1-R2-T2-W-F-16S**	167222	144052	141881	142027	104903	103762	62%
**M1-R3-T1-C-1-16S**	312758	190533	189598	189896	137226	131271	42%
**M1-R3-T1-C-2-16S**	970664	790233	788232	787627	567553	542254	56%
**M1-R3-T1-U-1-16S**	258843	207918	206916	207057	163422	160840	62%
**M1-R3-T1-U-2-16S**	363985	246672	246012	245346	188348	186443	51%
**M1-R3-T1-P-C-16S**	273822	222995	222050	222578	212140	211607	77%
**M1-R3-T1-P-F-16S**	96262	45382	44888	45091	39893	39840	41%
**M1-R3-T1-W-C-16S**	255147	202455	201319	201819	170404	169035	66%
**M1-R3-T1-W-F-16S**	517119	432405	429806	431440	384516	379034	73%
**M1-R3-T2-C-1-16S**	581936	493501	492383	492708	389446	368306	63%
**M1-R3-T2-C-2-16S**	425361	353832	353113	353240	271733	264471	62%
**M1-R3-T2-U-1-16S**	334565	266205	265596	265846	204210	199658	60%
**M1-R3-T2-U-2-16S**	391503	308982	308026	308294	236878	231449	59%
**M1-R3-T2-P-C-16S**	302462	240121	238983	239371	213719	211968	70%
**M1-R3-T2-P-F-16S**	262400	23372	22643	22989	21584	21488	8%
**M1-R3-T2-W-C-16S**	252945	205155	204083	204870	128835	128619	51%
**M1-R3-T2-W-F-16S**	397763	312142	310410	311526	265359	264191	66%
**M1-R4-T1-C-1-16S**	461871	391647	391038	391044	315181	294626	64%
**M1-R4-T1-C-2-16S**	743702	634522	633195	633064	490760	441999	59%
**M1-R4-T1-U-1-16S**	410114	347236	345941	345764	294687	288182	70%
**M1-R4-T1-U-2-16S**	579894	480455	478718	478612	406843	401851	69%
**M1-R4-T1-P-C-16S**	542846	435480	434246	434276	387267	383259	71%
**M1-R4-T1-P-F-16S**	407146	315326	313197	314284	288554	284710	70%
**M1-R4-T1-W-C-16S**	346834	286409	284936	285487	204431	203339	59%
**M1-R4-T1-W-F-16S**	280841	234465	231528	233030	205443	204412	73%
**M1-R4-T2-C-1-16S**	768437	674416	673522	673901	545930	508096	66%
**M1-R4-T2-U-1-16S**	400751	326204	325249	325220	265469	261315	65%
**M1-R4-T2-U-2-16S**	413440	339384	338014	338467	265937	261801	63%
**M1-R4-T2-P-C-16S**	419950	352357	351609	351949	332789	331917	79%

**M1-R4-T2-P-F-16S**	1558165	1267723	1262107	1266263	1131827	1121758	72%
**M1-R4-T2-W-C-16S**	489752	395719	394385	394903	338436	336616	69%
**M1-R4-T2-W-F-16S**	353823	288667	286427	287669	248425	246136	70%
**M1-BLANK-16S**	152760	130281	129501	129916	78068	77181	51%

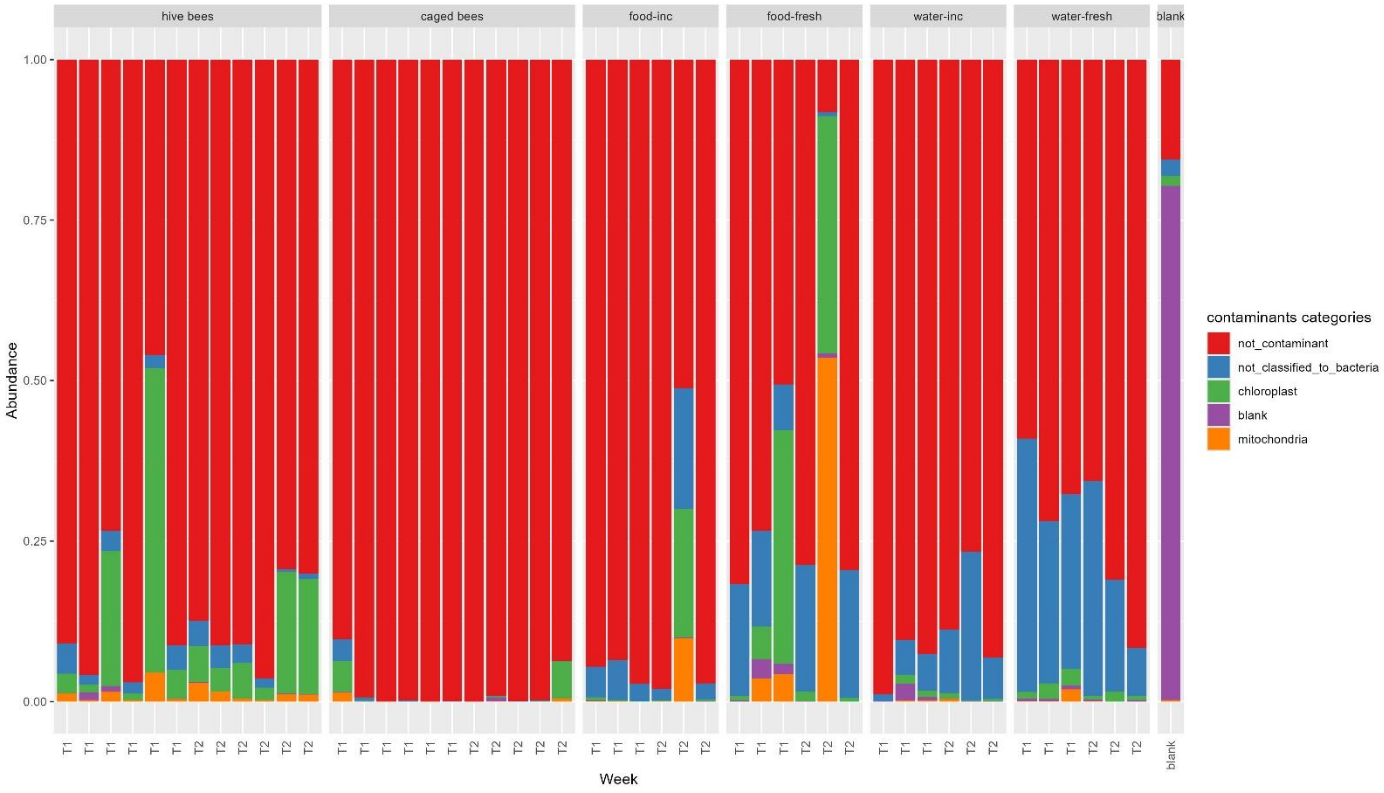

Fig. S2. Contaminant sources in the samples. Labels: 'inc' = incubated 24 h, 'fresh' = freshly prepared. ASVs not identified as contaminants are shown in red. Contaminant categories: ASVs not classified as bacteria (blue), ASVs identified as chloroplasts (green) or mitochondria (orange), ASVs identified in the blank sample and not reaching at least 10× of the abundance in the blank sample in any of the samples of interest (purple).

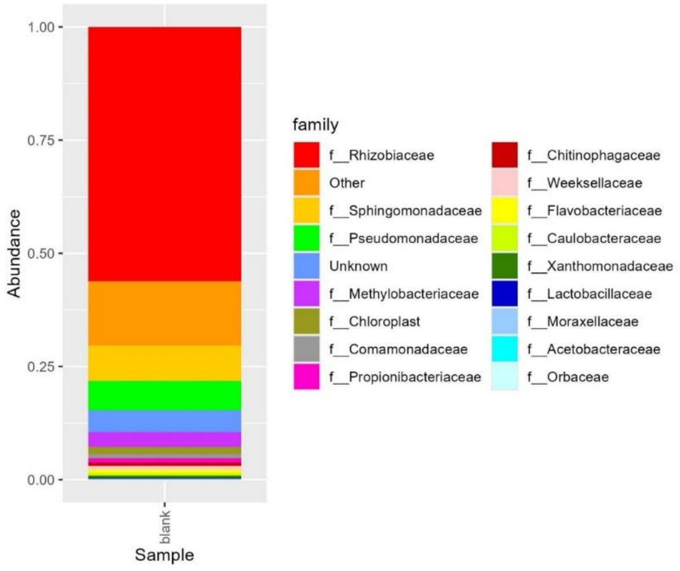

Fig. S3. Composition of the blank sample aggregated to family level. *Rhizobiaceae* dominated in the bacteria identified in the blank sample.

Table S3. Betadispersion tests results. Dispersion within bee sources was compared between hive bees and caged bees (a) and the distances of individual samples to each of the sample groups (hive bees, caged bees, water, food) were calculated (b).

a. **ANOVA test results for the betadispersion between hive bees and caged bees**. The dispersion differed significantly between bee sources.

	Df	Sum of squares	Mean square	F value	Pr(>F)
**source**	1	0.30910	0.309098	25.874	4.887e-05
**residuals**	21	0.25088	0.011946		

b. **Distances from group medians for the caged bees and hive bees**. Group – bee source, Nearest – nearest group (as for distance to group median) for each sample, Hive bees, Caged bees, Food, Water – distances to the respective group medians. Only the bee samples are shown. For all hive bees, the nearest spatial median was in their own group, for caged bees – three samples were closer to other groups' medians: two were nearest to the food group, one – to the hive bees' group.

SAMPLE	Group	Nearest	Hive bees	Caged bees	Food	Water
**M1-R2-T2-C-1-16S**	caged bees	caged bees	0.60256006	0.37977856	0.752374	0.690532
**M1-R2-T2-C-2-16S**	caged bees	caged bees	0.40037839	0.377063452	0.80708	0.765518
**M1-R3-T1-C-1-16S**	caged bees	caged bees	0.55283815	0.486626523	0.829322	0.769651
**M1-R3-T2-C-1-16S**	caged bees	caged bees	0.69236295	0.476372831	0.709695	0.686589
**M1-R3-T2-C-2-16S**	caged bees	caged bees	0.45391453	0.293851242	0.777447	0.748307
**M1-R4-T1-C-1-16S**	caged bees	caged bees	0.56103717	0.501654495	0.808537	0.751102
**M1-R4-T1-C-2-16S**	caged bees	caged bees	0.49775313	0.450385781	0.851222	0.804506
**M1-R4-T2-C-1-16S**	caged bees	caged bees	0.55109793	0.447214854	0.838293	0.784901
**M1-R2-T1-C-1-16S**	caged bees	food	0.79967589	0.63740798	0.399741	0.72578
**M1-R2-T1-C-2-16S**	caged bees	food	0.90293633	0.668151917	0.363954	0.694537
**M1-R3-T1-C-2-16S**	caged bees	hive bees	0.45237491	0.46370155	0.791614	0.748786
**M1-R2-T1-U-1-16S**	hive bees	hive bees	0.16050367	0.421648574	0.811591	0.763892
**M1-R2-T1-U-2-16S**	hive bees	hive bees	0.20060154	0.428862152	0.84255	0.785119
**M1-R2-T2-U-1-16S**	hive bees	hive bees	0.16902557	0.405786123	0.788493	0.735556
**M1-R2-T2-U-2-16S**	hive bees	hive bees	0.37354983	0.449605516	0.822265	0.76786
**M1-R3-T1-U-1-16S**	hive bees	hive bees	0.28145097	0.506170236	0.820999	0.770619
**M1-R3-T1-U-2-16S**	hive bees	hive bees	0.17686511	0.376492756	0.808395	0.752359
**M1-R3-T2-U-1-16S**	hive bees	hive bees	0.24928714	0.491869421	0.853406	0.803815
**M1-R3-T2-U-2-16S**	hive bees	hive bees	0.24926602	0.505501058	0.852699	0.802805
**M1-R4-T1-U-1-16S**	hive bees	hive bees	0.19326881	0.366431994	0.835632	0.781275
**M1-R4-T1-U-2-16S**	hive bees	hive bees	0.50437712	0.561917119	0.804451	0.735602
**M1-R4-T2-U-1-16S**	hive bees	hive bees	0.19375554	0.312876399	0.766463	0.709456
**M1-R4-T2-U-2-16S**	hive bees	hive bees	0.25686416	0.337762925	0.839558	0.787336

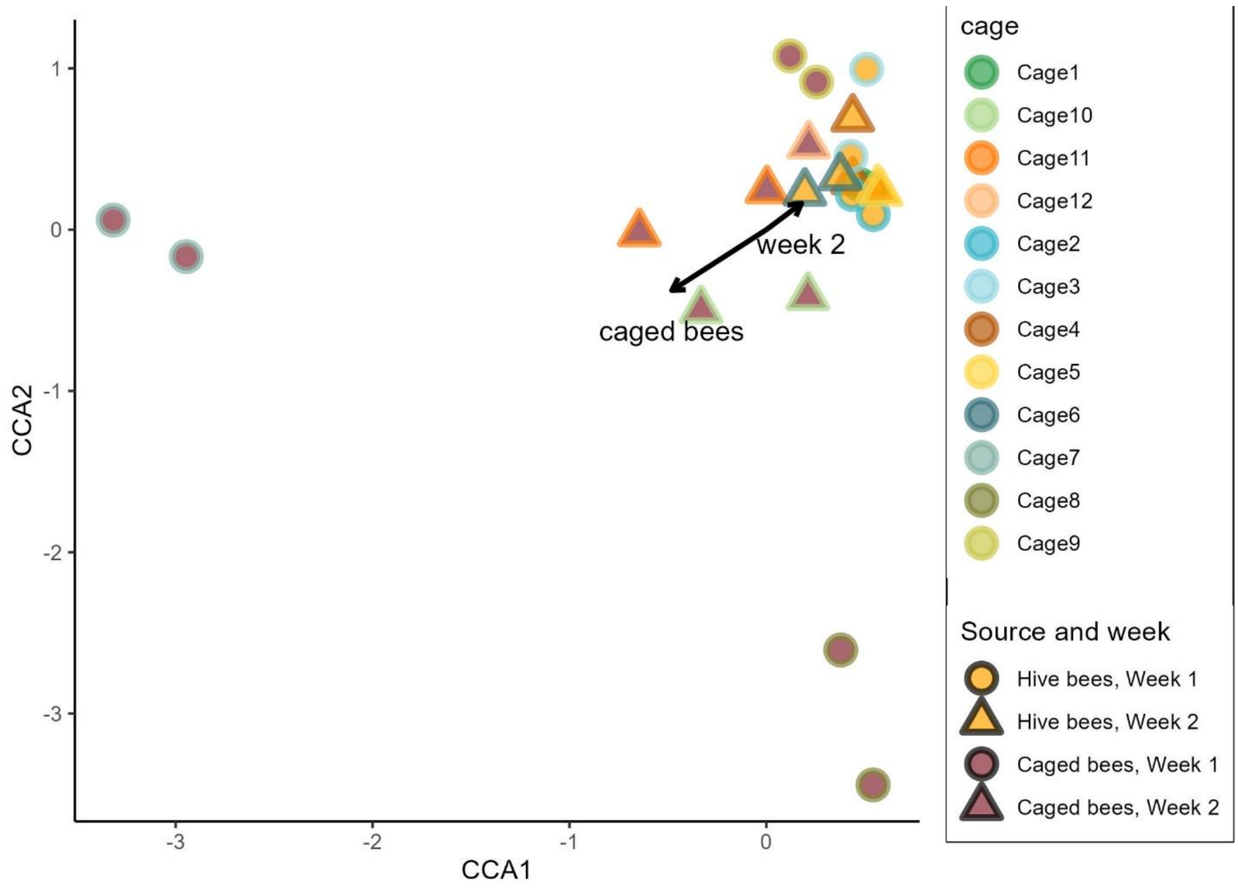

Fig. S4. CCA plot showing effects of bee source, timepoint, and cage on microbial community composition. CCA1 and CCA2 explain 61% and 41% of the constrained variance, respectively. Cage-specific axes are omitted for clarity. This figure is supplementary to Fig. 3a in the manuscript, with additional coloring of point contours by cage.

Table S4. CCA model summary for bees' samples. The following factors were included in the model: bee source (hive vs. caged bees), timepoint (week one and week two), and cage. The model explained 69% of the observed variance.

a. Model summary

	Inertia	Proportion	Rank
Total	3.2682	1.0000	
Constrained	2.2609	0.6918	11
Unconstrained	1.0072	0.3082	11

b. Eigenvalues for constrained axes. Some constraints or conditions were aliased because they were redundant.

CCA1	CCA2	CCA3	CCA4	CCA5	CCA6	CCA7	CCA8	CCA9	CCA10	CCA11
0.6091	0.4106	0.2975	0.2541	0.2225	0.1330	0.1066	0.1005	0.0609	0.0420	0.0241

c. Eigenvalues for unconstrained axes:

CA1	CA2	CA3	CA4	CA5	CA6	CA7	CA8	CCA9	CCA10	CCA11
0.26515	0.19416	0.12287	0.10361	0.09172	0.08002	0.05877	0.04127	0.02290	0.01443	0.01231

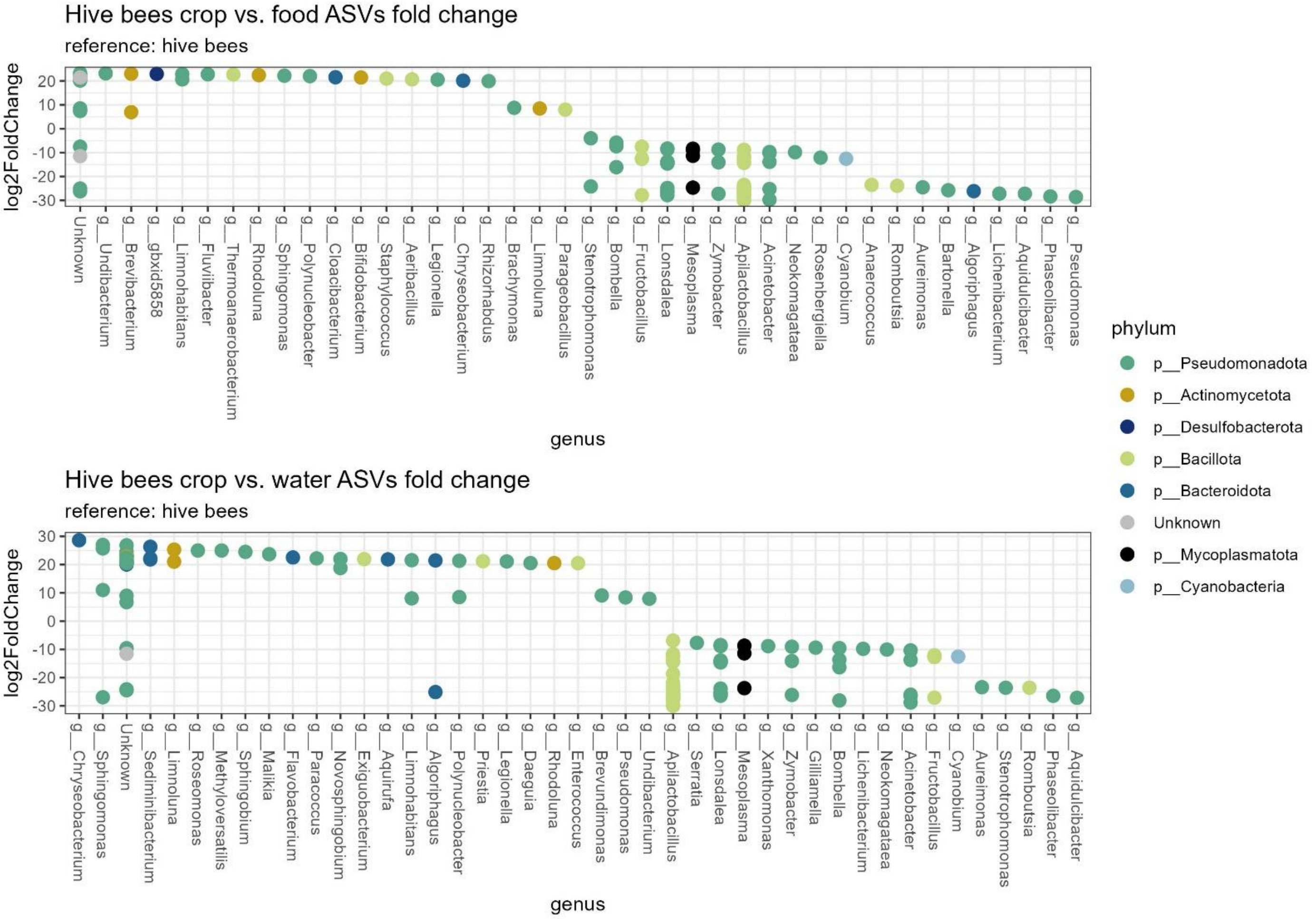

Fig. S5. Differential abundance analysis of bacterial ASVs between hive bees vs. food samples (top panel) and water samples (bottom panel). Only ASVs with significant differential abundance are shown (FDR < 0.01). Positive values indicate ASVs more abundant in food (34 ASVs) or in water (52 ASVs) in comparison to hive bees crop samples.
